# Recycling Black Tea Waste Biomass as Activated Porous Carbon for Long Life Cycle Supercapacitor Electrodes

**DOI:** 10.3390/ma14216592

**Published:** 2021-11-02

**Authors:** Hojong Eom, Jooyoung Kim, Inho Nam, Sunyoung Bae

**Affiliations:** 1 School of Chemical Engineering and Materials Science, Department of Intelligent Energy and Industry, Department of Advanced Materials Engineering, Chung-Ang University, Seoul 06974, Korea; ehj5738@cau.ac.kr; 2 Department of Chemistry, Seoul Women’s University, Seoul 01797, Korea; kjy3775@naver.com

**Keywords:** black tea carbon, biomass, energy storage materials, supercapacitors

## Abstract

Value creation through waste recycling is important for a sustainable society and future. In particular, biomass, which is based on crops, is a great recyclable resource that can be converted into useful materials. Black tea is one of the most cultivated agricultural products in the world and is mostly discarded after brewing. Herein, we report the application of black tea waste biomass as electrode material for supercapacitors through the activation of biomass hydrochar under various conditions. Raw black tea was converted into hydrochar via a hydrothermal carbonization process and then activated with potassium hydroxide (KOH) to provide a large surface area and porous structure. The activation temperature and ratio of KOH were controlled to synthesize the optimal black tea carbon (BTC) with a large surface area and porosity suitable for use as electrode material. This method suggests a direction in which the enormous amount of biomass, which is simply discarded, can be utilized in the energy storage system. The synthesized optimal BTC has a large surface area of 1062 m^2^ and specific capacitance up to 200 F∙g^−1^ at 1 mV∙s^−1^. Moreover, it has 98.8% retention of charge–discharge capacitance after 2000 cycles at the current density of 5 A∙g^−1^.

## 1. Introduction

Recently, there has been growing interest in converting and recycling daily-life waste into valuable materials. Tea is the most consumed flavored beverage in the world, and its cultivation is widespread. In particular, black tea accounts for approximately 78% of worldwide tea consumption and produces significant waste after brewing [1,2]. However, an enormous amount of black tea waste is generally discarded, even though it can be used as a biomass source. Biomass, which is based on crops that are generally used for food and beverages, is one of the most popular renewable resources [3,4,5]. It has been recycled into various renewable products such as bio-oils [6,7], bioplastic [8,9,10], biochemicals [11,12], and adsorbents [13], to date.

This study aimed to convert black tea waste into a valuable substance for energy storage systems. Demand for energy storage devices such as batteries and supercapacitors has been increasing over the past decade. Several biomasses have been applied to various types of energy storage devices, including fuel cells [14], Li-ion batteries [15], and supercapacitors [16,17]. Among them, supercapacitors are highlighted as an energy storage system because they have a long cycling life, rapid charge–discharge rates, cycle stability, and high power density. Therefore, we used black tea carbon (BTC) as a high-performance supercapacitor electrode with a large surface area and residual functional groups. Biomass has been known as a suitable carbon precursor with high porosity with conductivity [18] and many researchers have studied biomass-derived energy storage systems [19]. Unlike previous research that focused on the electric double-layer (EDL) capacitive effect of biomass-derived carbons [20,21,22], BTC has the potential to obtain large surface functional groups and a high pseudocapacitance effect. In this study, the effects of the physical and electrochemical properties of BTCs according to the activation temperature and the ratio of potassium hydroxide (KOH) were analyzed. Black tea biomass was carbonized to hydrochar through hydrothermal synthesis for effective electrode materialization. To obtain more porosity and a larger surface area, hydrochar was activated by KOH. The BTCs had a roughened surface and porous structure. Therefore, the original aspects of our research are as follows: (1) This study is based on specific black tea wastes. The consumed black tea wastes are greater than that of other types of tea (78%); (2) The synthesized material (BTCs) has a large surface functional group based on the residue of black tea. Therefore, we have predominantly shown pseudocapacitance effects. The two factors have not appeared in other studies to date.

We determined the optimal activation condition of BTCs, through physical properties including the surface area, residual functional groups, and electrochemical analysis to evaluate the electrical performance of the BTCs. To enhance the pseudocapacitance effect, we controlled the carbonization temperature and KOH ratio of BTCs, and the optimum point was 600 °C with a mass ratio of 1:3 between hydrochar: KOH. BTC that has an amorphous nature and large surface area can be easily utilized as electrode material. The maximum specific capacitance was 200 F∙g^−1^ at the scan rate of 1 mV∙s^−1^ and showed outstanding electrochemical cycle stability with 98.8% initial capacitance being retained after 2000 cycles at a current density of 5 A∙g^−1^. These properties possibly enabled the BTCs to act as a new biomass source of functionalized carbonaceous materials for high-performance supercapacitors.

## 2. Materials and Methods

### 2.1. Chemicals and Reagents

KOH was purchased from Ducksan Reagent (Ansan, Korea). Distilled water and pure water were used for the experiments (18.2 MWΩ∙cm, PURE ROUP 50, Purewater, Namyangju, Korea). A syringe filter with a pore size of 0.2 μm (polytetrafluoroethylene (PTFE) with a glass-fiber pre-filter, 13 mm ID, Echromscience, Daegu, Korea) and a filter paper with a pore size of 5–8 μm (No.20, Hyundai Micro., Seoul, Korea) were used for filtration.

### 2.2. Generation of Hydrochar from Black Tea Sludge

The different types of black tea leaves used in this study were purchased from the local market. These tea leaves were mixed evenly and then separated into small amounts to brew in distilled water at 92 °C for 4 min. This temperature and time were selected according to brewing instructions suggested by manufacturers. After brewing, the teas were filtered through a 250 μm sieve and kept refrigerated until they were used in the experiment. The water content of the above-treated black tea leaves was measured at 82.1% (±0.6). Carbonization was conducted without drying process.

The hydrothermal reaction conditions were selected from the previous study [23]. Black tea leaves (45 g) were transferred to the homemade reactor and allowed to react at 200 °C for 12 h. After 30 min of water cooling, the reactor at the end of the process measured the volume of the gas produced and pressure filtered to separate the hydrochar. At this time, the filtered liquid was bio-liquid, thus the mass was measured separately. The separated hydrochar was stirred for 24 h with 50 mL of tertiary distilled water, then decompressed and separated again. The obtained hydrochar was dried in the oven at 105 °C for 24 h, and stored in the desiccator until further experiments were required.

Using the following equation, the yield of hydrochar was calculated on a dry basis.

(1) $$\mathrm{Hydrochar}\;\mathrm{yield}\;(\%)=\frac{Solid\;content\;of\;hydrochar\;\left(g\right)}{solid\;content\;of\;feedstock\;\left(g\right)}\times 100\;(\%)$$

### 2.3. Generation of BTC

To enhance the performance of activated carbon as an electrode material, it was produced by activating hydrochar with KOH. The performance of activated carbon was evaluated at different hydrochar: KOH ratios of 1:2, 1:3, and 1:4 (*w*/*w*) and activation temperatures (600 °C and 700 °C). Hydrochar at 0.8 g and KOH were weighed in proportions of 1:2, 1:3, and 1:4, respectively, placed in the alumina crucible, and activated for 1 h at 600 and 700 °C under an air atmosphere using the electrical muffle space (Labtech, Namyangju, Korea). The obtained activated carbon was washed with hot, deionized, distilled water until the pH reached 7; then, it was pressure-reduction filtered, and dried in an oven at 105 °C for 24 h. The dried activated carbon was stored in a desiccator until it was used in further experiments. Each BTC was denoted as BTC-XY where X is the activation temperature, that is, X = 6 for 600 and 7 for 700 °C and Y is the mass ratio between hydrochar and KOH (12–14).

### 2.4. Structural Characterization

The morphology of the materials was confirmed by scanning electron microscopy (SEM) and transmission electron microscopy (TEM) images, which were obtained using a Carl Zeiss SIGMA field-emission scanning electron microscope and a JEOL JEM-F200 microscope, respectively. X-ray diffraction (XRD) patterns were evaluated by the Bruker New D8-Advance, which operated at 40 kV and 40 mA using CuKa radiation (1.5406 Å), to verify the crystalline part of the materials. To identify the components of each sample, X-ray photoelectron spectroscopy (XPS) was conducted using the Thermo Fisher Scientific K-alpha + instrument that set the carbon peak as standard (C 1 s = 284.5 eV). The surface area was measured from the BET nitrogen adsorption/desorption isotherms using a Micromeritics 3Flex analyzer. The pore volume and diameter were calculated by exploring the desorption of the isotherm using the Barrett–Joyner–Halenda (BJH) method. The functional groups and binding of elements were analyzed by Fourier-transform infrared (FT-IR) spectroscopy using a Thermo Scientific (Waltham, MA, USA) Nicolet 6700 spectrometer. 

### 2.5. Electrochemical Analysis

Electrochemical analyses were conducted under a standard three-electrode system. A working electrode was combined with active materials (80 wt%), carbon black (Super-P, 10 wt% from Hyundai, Seoul, Korea), and PTFE (10 wt%, from Soulbrain, Seongnam, Korea). After mixing these components, isopropanol was added to make the electrode dough. This was then coated on the attached stainless SUS mesh (1 cm^2^) and compressed with a hydraulic compressor to minimize the resistance. The platinum electrode was used as a counter electrode, and a NaCl-saturated Ag/AgCl electrode was used as a reference electrode, while a 1 M H_2_SO_4_ solution was used as an electrolyte. 

The electrochemical workstation (ZIVE SP1, WonA Tech, Seoul, Korea) was used for cyclic voltammetry (CV), galvanostatic charge/discharge (GCD), and electrochemical impedance spectroscopy (EIS) analyses. CV was conducted in a voltage range of −0.2–0.8 V while varying the scan rate in the range of 1–100 mV∙s^−1^. The GCD was performed on various current densities ranging from 0.5 to 5 A∙g^−1^ within the potential voltage range of −0.2–0.8 V. The EIS frequency range was 1–10 MHz at an applied potential of 0.5 V.

## 3. Results

### 3.1. Physiochemical Characterization

**Figure 1:**
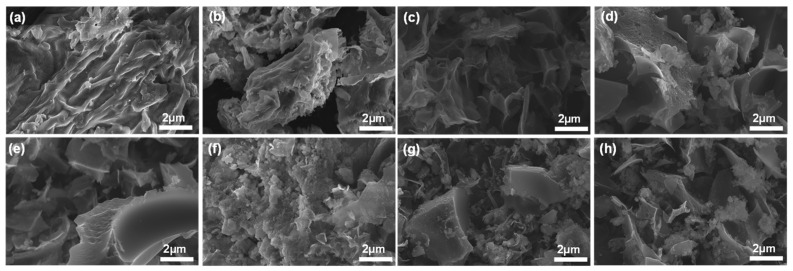
SEM images of (**a**) raw black tea, (**b**) hydrochar, (**c**) BTC-612, (**d**) BTC-613, (**e**) BTC-614, (**f**) BTC-712, (**g**) BTC-713, and (**h**) BTC-714 (denoted as BTC-XY where X indicates the activation temperature, that is, 6 for 600 °C and 7 for 700 °C and Y is the mass ratio between hydrochar and KOH).

The external morphologies of the raw black tea, hydrochar, and BTC were analyzed using SEM images (Figure 1). As mentioned earlier, each BTC was denoted as BTC-XY; where X is the activation temperature, and Y is the mass ratio between hydrochar and KOH. Before being activated with KOH, both raw black tea and hydrochar had smooth surfaces with wavy patterns. However, under every activation condition, a porous structure was indicated on the surface of BTC. At the same activation temperature, as the ratio of KOH increased, the BTCs exhibited a more porous and rougher surface. Even at a lower temperature of 600 °C, most of the fibrous structures of black tea disappeared. All activation conditions showed irregularly activated pore sizes in a random position.

**Figure 2:**
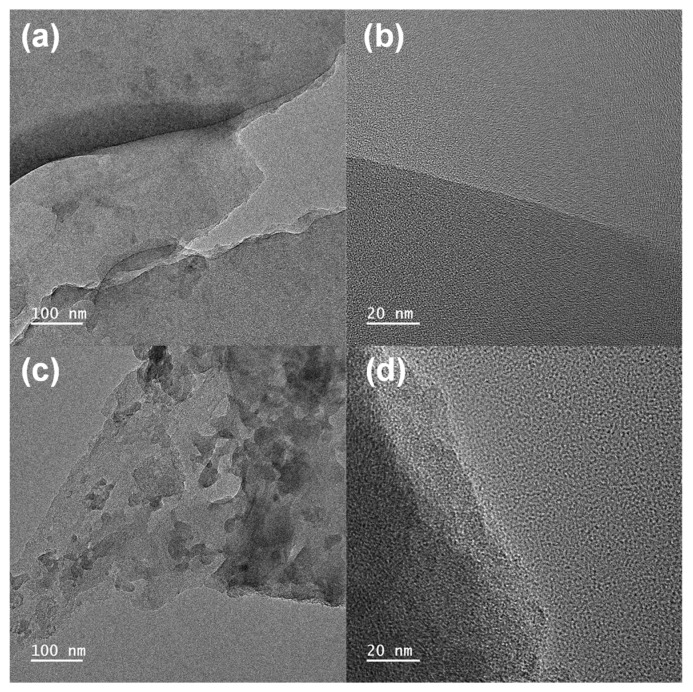
TEM images of BTC at (**a**,**b**) 600 °C and (**c**,**d**) 700 °C.

FE-TEM was conducted to determine the internal pore structure of the BTC (Figure 2). The TEM images showed the porous structure and rough surface caused by the activation process. No significant and noticeable difference was observed in each activation condition; however, during activation, KOH formed a continuous micropore inside a BTC that provided space for electrolyte penetration and maximized the surface area. At high resolutions, no crystalline parts were found, which indicates that the BTC has an amorphous structure with some graphitic structure [24].

**Figure 3:**
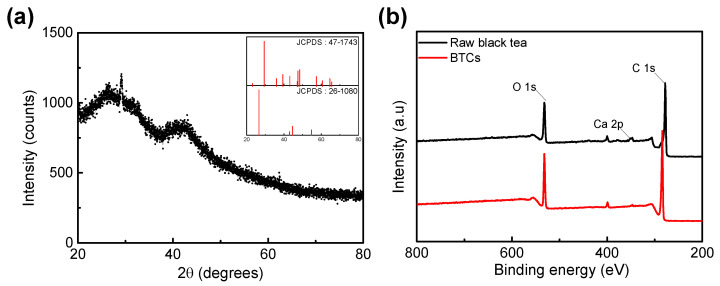
(**a**) XRD spectrum BTC (Inset: JCPDS patterns of CaCO_3_ (No. 47–1743) and carbon (No. 26-1080)). (**b**) XPS spectrum of raw black tea and BTCs.

This is in good correspondence with the XRD data (Figure 3a). The XRD patterns of the BTC exhibited two broad peaks at 23° and 43°. The broad peaks correspond to amorphous carbon with a vaguely graphitic structure [25]. However, a sharp crystalline peak corresponding to CaCO_3_ was observed at 29° [26]. Black tea is rich in minerals such as Ca [27]. JCPDS patterns of CaCO_3_ (No. 47–1743) and carbon (No. 26–1080) were also plotted in the inset of Figure 3a, which is well correlated with the XRD pattern. Furthermore, the XPS spectrum of the raw black tea and BTCs confirmed the presence of Ca. In Figure 3b, the total range of XPS spectrum, the peaks that correspond to the Ca 2p were detected in both raw black tea and BTC. CaCO_3_ is an inactive component for faradaic reaction in this voltage range [28], so it might be the reason for increasing the resistance of the electrode [29]. However, the amount of Ca contained in BTC is negligible (0.32%), so the effect due to CaCO_3_ seems to be insignificant. 

**Figure 4:**
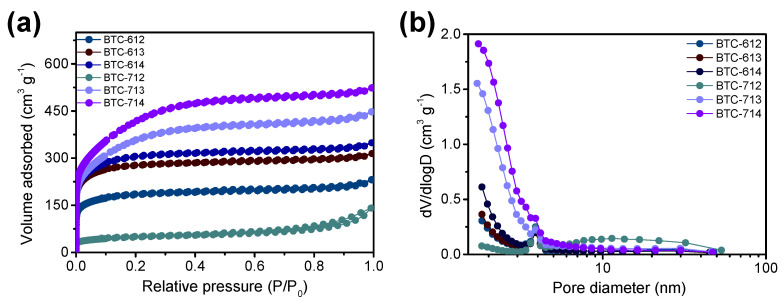
(**a**) N_2_ adsorption/desorption isotherms and (**b**) pore size distribution of the BTCs.

The N_2_ adsorption/desorption isotherms and the BJH method’s pore-size distribution were analyzed from the desorption pattern of the N_2_ isotherms for the BTCs in Figure 4. The micropore volume was estimated by a *t*-plot method (Table 1). The shapes of all the observed plots resemble a mixture of Type I isotherms as prescribed by the International Union of Pure and Applied Chemistry (IUPAC), which is widely observed in microporous developed materials, particularly in activated carbon. The observed pore size distribution demonstrates the existence of micropores and mesopores in all the BTCs; however, there is no ordered pore structure. It was found that the surface area and micropore volume of the BTC increase as the ratio of KOH increases, which can enlarge the capacitance of BTC.

**Figure 5:**
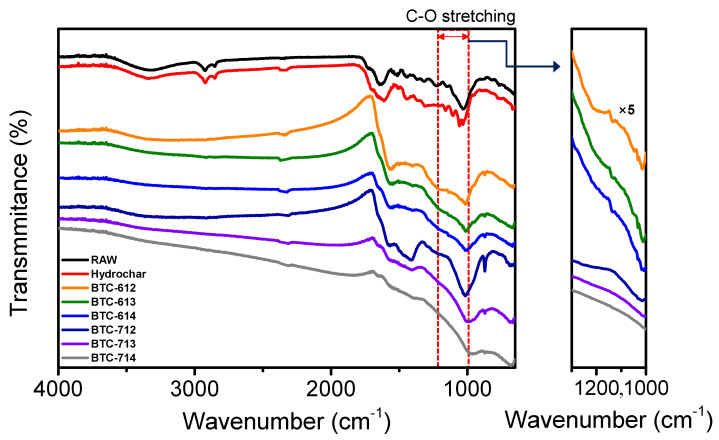
FT-IR spectrum of BTCs.

FT-IR was used to indicate and compare the residual functional groups of the raw black tea, hydrochar, and BTC which were activated in various conditions (Figure 5). The peak between 3600 and 3000 cm^−1^ represents OH stretching such as alcohol and carboxylic acid. The small peaks at 2918 cm^−1^ and 2850 cm^−1^ represent the C-H asymmetric and symmetric stretching of the methylene and methyl groups, while the peak at 2850 cm^−1^ correlates with the –CH stretching of the cellulose backbone. The absorption bands at 1612 cm^−1^ and 1547 cm^−1^ were assigned C=C stretching and aromatic vibrations, respectively. After activation, every peak was similarly weakened or disappeared under all conditions. However, the range from 1300 cm^−1^ to 1000 cm^−1^, which corresponds to C-O stretching, showed a different tendency. The peak, which was strong in raw black tea and hydrochar, weakened slightly at 600 °C and completely disappeared at 700 °C. Moreover, this shows that the peak became weaker as the ratio of KOH increased at the same temperature. This means that when using BTC as a pseudocapacitor material, the remaining functional groups at 600 °C activation can act more effectively. Based on the data to date, BTC has a large surface area, but most of these surfaces are micropores, so it is difficult for diffusion, and the internal path is not visible, so it is appropriate to use it as a pseudocapacitor rather than EDLC. Therefore BTC, which has more residual functional groups and a large surface area, will enhance the characteristics of pseudocapacitors.

### 3.2. Electrochemical Performance

**Figure 6:**
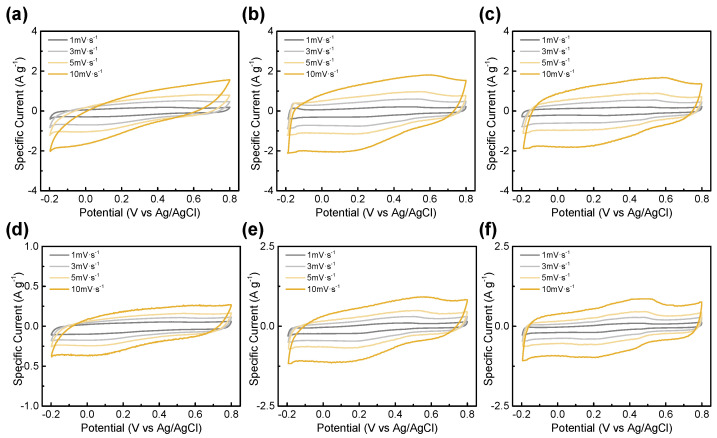
Cyclic voltammograms of (**a**) BTC-612, (**b**) BTC-613, (**c**) BTC-614, (**d**) BTC-712, (**e**) BTC-713, and (**f**) BTC-714 at scan rate from 1 to 10 mV·s^−1^.

The electrochemical properties of the BTCs with different hydrochar: KOH mass ratios at different activation temperatures were investigated using CV, GCD, and EIS in a three-electrode configuration. All electrochemical experiments were conducted in 1 M H_2_SO_4_ acidic electrolyte at room temperature. Figure 6 shows the CV curves of the BTCs with different activated conditions, and the curves were measured at a scan rate of 1, 3, 5 and 10 mV∙s^−1^ in the voltage window of −0.2 V to 0.8 V versus an Ag/AgCl reference electrode. The BTC electrodes presented a box-shaped pseudocapacitive reaction at all scan rates, which indicates that these samples had a good rate capability. At the activation temperature of 600 °C, as the KOH ratio increases, the I–V graphs become more rectangular. However, if the activation temperature increases to 700 °C, the area of the I–V graph becomes smaller, which is due to the less faradaic reaction by the residual functional group. No faradaic reaction caused by the functional group decreases specific capacitance. This is also consistent with BTC-613 and BTC-713 having the largest surface areas with functional groups. There is no significant difference in surface area. The capacitance generated by the faradaic reaction of more residual functional groups expands the I–V graph area and total specific capacitance.

**Figure 7:**
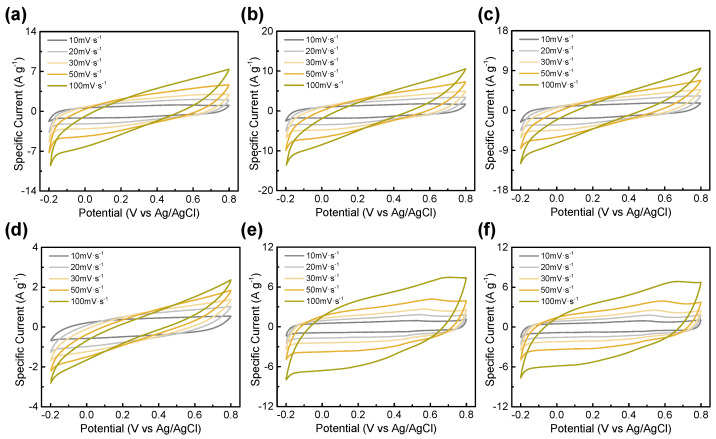
Cyclic voltammograms of (**a**) BTC-612, (**b**) BTC-613, (**c**) BTC-614, (**d**) BTC-712, (**e**) BTC-713, and (**f**) BTC-714 at scan rate from 10 to 100 mV·s^−1^.

The specific capacitances of the materials were calculated from the CV response at different scan rates using the following equation [30]:

(2) $$C_{\mathrm{sp}}=\frac{1}{v\;\left(V_{2}-V_{1}\right)}\;\int_{V_{1}}^{V_{2}}I\left(V\right)\mathrm{dV}$$

where C_sp_, *v*, V_1_, V_2_, and I(V) are the specific capacitance, scan rate, discharge voltage limit, charge voltage limit, and voltammogram current density (A∙g^−1^), respectively. The specific capacitances of BTC-612, BTC-613, and BTC-614 at 1 mV·s^−1^ were 148, 200, and 159 F·g^−1^, respectively. Moreover, those of BTC-712, BTC-713, and BTC-714 at 1 mV·s^−1^ were 53, 110, and 97 F·g^−1^, respectively. BTC-613 showed the highest specific capacitance at all scan rates. The CV curves measured at a scan rate from 10 mV·s^−1^ to 100 mV·s^−1^ are shown in Figure 7. As the scan rate increases, the box-shaped graph tends to be distorted in BTC-600 °C. However, they still maintained a bigger capacitance than BTC-700 °C. As mentioned earlier, this is related to the faradaic reaction. BTC-614, BTC-713, and BTC-714 had a larger specific surface area than BTC-613; however, the difference in the surface areas is insignificant. Therefore, the effect of the faradaic reactions caused by the more residual functional group would have been greater than that of the capacitance.

**Figure 8:**
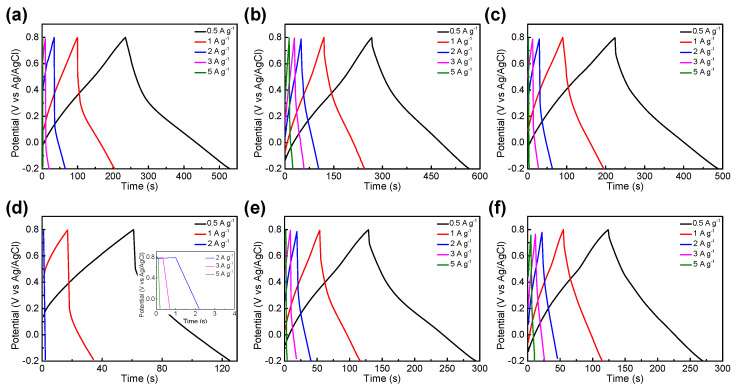
The galvanostatic charge–discharge curves for (**a**) BTC-612, (**b**) BTC-613, (**c**) BTC-614, (**d**) BTC-712, (**e**) BTC-713, and (**f**) BTC-714.

**Figure 9:**
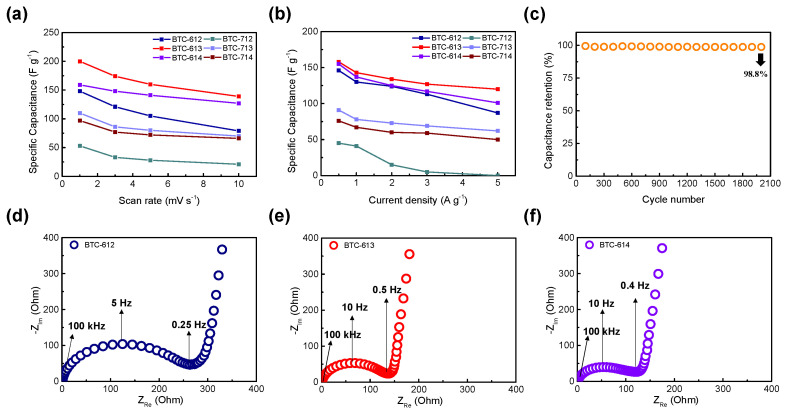
(**a**) Specific capacitance of BTCs at various scan rates ranging from 1 to 10 mV∙s^−1^, (**b**) specific capacitance of BTCs at various current densities from 0.5 to 5 A∙g^−1^, (**c**) capacitance retention of BTC-613 electrode over 2000 cycles at a current density of 5 A∙g^−1^. (**d**) ElS plot of BTC-612, (**e**) BTC-613, (**f**) ElS plot of BTC-614.

Figure 8 presents the GCD graph of the BTC electrodes. Each electrode presented symmetric linearity but slightly dented slopes within the potential range of from −0.2 V to 0.8 V, which is a characteristic of a pseudocapacitor electrode [30]. BTCs activated at 600 °C showed longer charge–discharge times than BTCs activated at 700 °C. BTC-613 exhibited the longest charge–discharge time and a small internal resistance, indicating a good Coulombic efficiency during charge and discharge. The overpotential of supercapacitor materials can be indicated from the IR drop, obtained from GCD analysis, which is a potential decline due to the resistance [31,32]. The internal resistances (equivalent series resistance, R_ESR_) from overpotential by GCD for various materials were listed in Table 2. Additionally, BTC-613 has the highest specific capacitance value and energy density (Table 2) which agrees with CV data. The specific capacitance values of the electrodes with respect to the scan rates and current densities were calculated and plotted in Figure 9a,b. The R_ESR_ was calculated with the IR drop (ΔV) and the constant current density (I) based on the equation [33]:

$$R_{\mathrm{ESR}}=\frac{\Delta V}{2\cdot I}$$

BTC-613 has the smallest R_ESR_ (0.06 Ω) at 0.5 A·g^−1^ among 600 °C treated BTC, and a larger capacitance than BTC-714, which has a smaller R_ESR_ (0.056 Ω). The IR drop at the start point of the discharge curve signifies the resistance of the electrode materials and electrolytes. In general, the former contributes more to the overall IR drop.

EIS was conducted to identify the charge transfer processes and resistances in the material structure, and the corresponding Nyquist plots of BTCs activated at 600 °C are presented in Figure 9. EIS spectra were recorded in the frequency range of from 1 to 10 MHz at room temperature. The size of the semicircle signifies the charge transfer resistance (R_ct_), which is relevant to the sum of the contact resistance at the interface of the electrode and current conductor [34], faradaic reactions occurring in pseudocapacitive materials, electrolyte resistance at the interface of porous carbon [35], and electrode resistance [36].

BTC-612, BTC-613, and BTC-614 had R_ct_ values of 257, 138, and 133 Ω, respectively. All BTCs had higher resistance values than normal supercapacitors, which indicates that the charge-transfer resistance was significantly greater at the interface. BTC-614 had a slightly larger surface area and a smaller R_ct_ value compared to BTC-613. However, the effects of the residual functional group caused faradaic reactions, allowing BTC-613 to have a more specific capacitance. In the low-frequency region, the slopes were 5.49, 9.39, and 6.45, respectively. Additionally, Warburg impedance (Z_ω_) at a low frequency was calculated by the equation as follows [37]:

$$Z_{\omega }=\frac{\sigma_{\omega }\omega^{-0.5}}{\sqrt{2}}$$

where σ_ω_ is the Warburg coefficient, which can be obtained from the slope of the linear fitting of the real part of the impedance (Z_Re_) versus the square root of angular frequency (ω^−0.5^). Warburg impedance values of BTC-612, BTC-613, and BTC-614 are 83.5, 41.9, and 51.6 Ω, respectively. BTC-613 has a graph close to vertical and the smallest Warburg impedance value, which means it can be the ideal supercapacitor with fast ion diffusion.

The long cycle test was conducted to identify the cycle stability of the BTC electrode. It is an important factor for an energy storage system. As shown in Figure 9c, capacitance retention of BTC-613 was maintained over the 2000 cycles at a current density of 5 A·g^−1^. The energy loss of only 1.2% during the 2000 cycles indicates that BTC is appropriate to be utilized as electrode material.

Finally, the electrochemical performance of various biomass-based supercapacitor electrode materials is summarized to compare with BTC (Table 3). BTC shows good electrochemical performance compared to other biomass-based electrode materials and lies at a high level.

## 4. Conclusions

Hydrochar based on black tea waste biomass was activated at different temperature conditions and KOH ratios. Through activation, porous graphitic activated carbon was synthesized for use as an electrode material in supercapacitors. For the most optimal activation point, BTC-613 activated at 600 °C with a hydrochar: KOH ratio of 1:3, had a large surface area (1062 m^2^·g^−1^) and porous structure while maintaining more residual functional groups on the surface compared to BTCs activated under other conditions. Furthermore, BTC-613 shows enlarged electrochemical performance as an electrode material for pseudocapacitors. The residual functional groups caused a more faradaic reaction that induced a faradaic current. The BTC-based pseudocapacitor exhibited a specific capacitance of 200 F·g^−1^ at 1 mV·s^−1^ and performed with more than 98.8% cyclic stability in more than 2000 cycles. Hence, a simple method of fabricating activated carbon from black tea waste biomass for electrode materials could present new recycling sites for crop-based biomass and new pathways applicable to energy storage systems. On the other hand, when compared with conventional pseudocapacitor or EDLC materials, BTC is still insufficient in terms of electrochemical performance. There are two main challenges to commercializing this material: (1) the performance should be increased; (2) the yield of production should be increased. This paper focused on recycling black tea biomass as an energy storage electrode. Our next goal will be to improve the electrochemical performance of BTC compared to commercial materials with the same economic feasibility.

## Figures and Tables

**Table 1:** BET surface area and pore volume of the BTCs.

BTC-XY	BET Surface Area [m^2^∙g^−1^]	Total Pore Volume [cm^3^∙g^−1^]	Micropore Volume [cm^3^∙g^−1^]
BTC-612	693	0.3404	0.2783
BTC-613	1062	0.4755	0.4244
BTC-614	1137	0.5251	0.4641
BTC-712	174	0.1909	0.0516
BTC-713	1227	0.6754	0.5539
BTC-714	1398	0.7967	0.6893

**Table 2:** Electrochemical performance results of various BTC at a current density of 0.5 A·g^−1^.

BTC-XY	ESR [Ω]	Specific Capacitance [F·g^−1^]	Energy Density [Wh·kg^−1^]	Power Density [W·kg^−1^]
BTC-612	0.166	146	20.3	300
BTC-613	0.060	159	22.1	266
BTC-614	0.122	156	21.7	286
BTC-712	0.297	45	6.27	358
BTC-713	0.102	91	12.7	279
BTC-714	0.056	76	10.6	265

**Table 3:** The electrochemical performance of various biomass-based supercapacitors.

Materials	Specific Capacitance [F·g^−1^]	Measurement Condition	Reference
Black tea	200	1 mV·s^−1^/1 M H_2_SO_4_	This paper
Coffee	51	0.3 A·g^−1^/1 M Li_2_SO_4_	[38]
Green tea	135	0.5 A·g^−1^/1 M H_2_SO_4_	[39]
Tea leave	148	0.5 A·g^−1^/3 M LiOH	[21]
Tea waste	220	0.1 mV·s^−1^/1 M Na_2_SO_4_	[20]
Tea (Mao feng)	275	1 A·g^−1^/2 M KOH	[22]
Cotton	118	1 A·g^−1^/1 M TEABF_4_/AN	[40]
Coconut	91	0.2 A·g^−1^/1 M TEMABF_4_/PC	[41]
Eggshell	55	0.15 A·g^−1^/2 M NaOH	[28]

## Data Availability

Data Sharing is not applicable.
